# Predicting the Prognosis of Bladder Cancer Patients Through Integrated Multi-omics Exploration of Chemotherapy-Related Hypoxia Genes

**DOI:** 10.1007/s12033-024-01203-9

**Published:** 2024-05-28

**Authors:** Wensheng Shi, Jiaming Dong, Bowen Zhong, Xiheng Hu, Chunguang Zhao

**Affiliations:** 1 Hunan Key Laboratory of Skin Cancer and Psoriasis, Department of Dermatology, Hunan Engineering Research Center of Skin Health and Disease, Xiangya Hospital, Central South University, Changsha, 410008 Hunan China; 2 National Engineering Research Center of Personalized Diagnostic and Therapeutic Technology, Central South University, Changsha, 410008 Hunan China; 3 Furong Laboratory, Changsha, 410008 Hunan China; 4 Department of Urology, Xiangya Hospital, Central South University, Changsha, 410008 Hunan China; 5 Department of Radiation, Cangzhou Central Hospital, Hebei, 061000 China; 6 Department of Critical Care Medicine, National Clinical Research Center for Geriatric Disorders, Xiangya Hospital, Central South University, Changsha, 410008 China

**Keywords:** Bladder cancer, Hypoxia-related genes (HRGs), Tumor immune microenvironment (TIME), Prognosis, Chemotherapy, Doxorubicin

## Abstract

Bladder cancer is a prevalent malignancy with high mortality rates worldwide. Hypoxia is a critical factor in the development and progression of cancers. However, whether and how hypoxia-related genes (HRGs) could affect the development and the chemotherapy response of bladder cancer is still largely unexplored. This study comprehensively explored the complex molecular landscape associated with hypoxia in bladder cancer by analyzing 260 hypoxia genes based on transcriptomic and genomic data in 411 samples. Employing the 109 dysregulated hypoxia genes for consensus clustering, we delineated two distinct bladder cancer clusters characterized by disparate survival outcomes and distinct oncogenic roles. We defined a HPscore that was correlated with a variety of clinical features, including TNM stages and pathologic grades. Tumor immune landscape analysis identified three immune clusters and close interactions between hypoxia genes and the various immune cells. Utilizing a network-based method, we defined 129 HRGs exerting influence on apoptotic processes and critical signaling pathways in cancer. Further analysis of chemotherapy drug sensitivity identified potential drug–target HRGs. We developed a Risk Score model that was related to the overall survival of bladder cancer patients based on doxorubicin-target HRGs: ACTG2, MYC, PDGFRB, DHRS2, and KLRD1. This study not only enhanced our understanding of bladder cancer at the molecular level but also provided promising avenues for the development of targeted therapies, representing a significant step toward the identification of effective treatments and addressing the urgent need for advancements in bladder cancer management.

**Supplementary Information:**

The online version contains supplementary material available at 10.1007/s12033-024-01203-9.

## Introduction

Bladder cancer ranks among the top 10 most prevalent cancers worldwide, accounting for 200,000 fatalities and 550,000 new cases each year [1]. Bladder cancer represents a complex and heterogeneous malignancy, posing significant challenges in terms of understanding its underlying molecular mechanisms and identifying effective therapeutic strategies [2]. Hypoxia, characterized by insufficient oxygen supply, plays a pivotal role in shaping the tumor microenvironment and influencing gene expression patterns [3–5]. However, the specific involvement and characterization of HRGs in bladder cancer remain less explored. In this study, we aimed to investigate the relationship between transcriptomic and genomic perturbation of HRGs and bladder cancer, shedding light on the molecular mechanisms underlying tumor development.

Accumulating evidence has suggested that the tumor immune microenvironment (TIME) plays an essential role in bladder cancer development [6–8]. Notably, TIME can also influence the efficacy of chemotherapy for bladder cancer patients [9, 10]. We hypothesized that HRGs might not only impact the immune microenvironment but also influence the response to chemotherapy in bladder cancer. By integrating transcriptomic data with immune cell infiltration profiles, we sought to elucidate the correlations between HRGs, the immune landscape, and chemotherapy response in bladder cancer.

Identification of potential therapeutic targets is crucial for improving treatment outcomes in bladder cancer [11]. Given the emerging role of HRGs in tumor biology, we employed a network-based approach to identify HRGs that could serve as chemotherapy targets in bladder cancer [12, 13]. By constructing an integrated network encompassing gene expression data and drug-gene interactions, we aimed to prioritize HRGs with the potential to be targeted by existing chemotherapeutic agents.

In this study, we performed a comprehensive investigation into the role of 260 hypoxia genes in 411 TCGA BLCA samples, leveraging multi-omic data to decipher the intricate molecular landscape. We applied an unsupervised clustering method to identify the oncogenic roles and prognostic values of hypoxia genes in bladder cancer subtypes. This study also delved into the intricate relationship between hypoxia genes and the immune landscape, revealing unique correlations with specific immune cells. A network-based approach was employed to identify HRGs that contribute to drug resistance in bladder cancer. By comprehensively analyzing hypoxia genes and their impact on diverse aspects of bladder cancer biology, we aim to contribute valuable insights that may facilitate improvement in precision medicine and clinical management strategies in bladder cancer.

## Materials and Methods

### Collection of Hypoxia Gene Sets

A total of 272 hypoxia genes were obtained from the intersection of two gene sets in The Molecular Signatures Database (MSigDB) [14] database (https://www.gsea-msigdb.org/gsea/msigdb) including: (1) 200 genes from “HALLMARK HYPOXIA” set. (2) 75 genes from “REACTOME CELLULAR RESPONSE TO HYPOXIA” set.

### Transcriptomic and Genomic Profile Acquisition

The Fragments Per Kilobase of transcript per Million mapped reads upper quartile (FPKM-UQ) format of RNA-seq data for The Cancer Genome Atlas Urothelial Bladder Carcinoma (TCGA-BLCA) tumor (*n* = 411) and adjacent non-tumorous tissue (*n* = 19) were downloaded from UCSC Xena website [15] (https://xenabrowser.net/). The somatic mutation and copy number variation (CNV) profiles of BLCA patients were obtained from the TCGA Multi-Center Mutation Calling in Multiple Cancers (MC3) project [16]. 260 of 272 hypoxia genes from MSigDB were detected in the TCGA expression profile.

### Global Overview of Hypoxia Genes Perturbation in BLCA

Here, we performed a two-step analysis to provide an in-depth understanding of the hypoxia genes in BLCA using transcriptomic and genomic data.

1. Differential expression analysis was performed by R package ‘limma’ v 3.54.2 to identify expression perturbation in this study [17] based on transcriptomic profiles. Hypoxia genes with fold change > 1.5 and Benjamini–Hochberg (BH) adjusted *P* value < 0.05 were identified as dysregulated.
2. Genomic perturbation of hypoxia genes in BLCA patients was analyzed by identifying genes with high-frequency somatic mutation or CNV. R package ‘maftools’ v 2.14.0 was used to visualize the categories and frequency of hypoxia genes’ somatic mutation [18].

### Survival Analysis

The overall survival (OS), disease-free interval (DSS), and progression-free interval (PFI) were obtained from the USCS Xena platform [15]. We applied univariate and multivariate Cox regression model by R packages ‘survival’ v 3.5–7 and ‘survminer’ v 0.4.9 to identify genes with prognostic value [19, 20]. Genes with Cox significance *P* value < 0.05 were identified as prognostic genes. Among them, genes with Hazard Ratio (HR) > 1 were identified as risky genes, whereas genes with HR < 1 were protective genes. Kaplan–Meier (K–M) survival curves of the log-rank test were applied to compare the survival differences between two groups of patients.

### Identification of Two BLCA Clusters with Oncogenic Roles

We applied an unsupervised consensus clustering method by R package ‘ConsensusClusterPlus’ v 1.62.0 based on dysregulated hypoxia genes in tumor samples to define distinct BLCA clusters [21]. The optimal number of clusters from *k* = 2:8 was determined by Calinski criteria [22]. In order to characterize the oncogenic roles of BLCA subtypes, we collected 20 oncogenic gene sets from previous studies, including 10 sets from oncogenic pathways and 10 sets from cancer hallmark genes [23, 24]. Next, we performed Single-sample gene-set enrichment analysis (ssGSEA) to quantify the expression level of each gene set in BLCA samples based on the transcriptomic data [25]. Then, we compared the expression differences of 20 gene sets between distinct BLCA clusters.

### HPscore was Correlated with Clinical Features and Chemotherapy Responses

The clinical information including age, gender, survival status, diagnosis subtype, TNM stage, histological grade, and pathologic stage was obtained from the TCGA database [26]. We defined a HPscore using ssGSEA method based on the expression profiles of prognosis-related hypoxia genes in BLCA. Based on the median value of the HPscore, BLCA patients were divided into High/Low HP groups. To explore the correlations between HPscore and clinical features, we compared the HPscore differences between distinct clinical groups by Mann–Whitney *U* test (two groups) and Kruskal–Wallis test (more than two groups) [27–29]. The chemotherapy response information of patients was collected from the TCGA database [26]. Patients were divided into responders (complete or partial response to chemotherapy) and non-responders (stable or progressed disease after chemotherapy). The HPscore differences between responders and non-responders were compared by the Mann–Whitney *U* test and Kruskal–Wallis test.

### HPscore was Correlated with TIME in BLCA

We collected a transcriptomics-based TIME signature including 29 features from a previous study [30]. The immune cell abundance, stromal cell abundance, tumor purity, and overall TIME score of BLCA were generated by the Estimation of Stromal and Immune cells in Malignant Tumor tissues using Expression data (ESTIMATE) method [31]. First, the expression level of 29 features in BLCA tumor samples was quantified by the ssGSEA method. Next, we performed an unsupervised hierarchical clustering analysis based on TIME features on BLCA samples. Then, we performed the fisher test to explore the overlap among BLCA consensus clusters, HP groups, and immune clusters, respectively. The immune cell abundances were collected from Tumor IMmune Estimation Resource (TIMER) v2.0 database (http://timer.cistrome.org/) [32–34]. The marker genes of macrophages in BLCA were collected from the CellMarker v2.0 database (http://bio-bigdata.hrbmu.edu.cn/CellMarker) [35]. A co-expression network between hypoxia genes and marker genes of various immune cells was constructed by Pearson’s correlation analysis.

### Identification of HRGs in BLCA

We performed a three-step analysis to identify HRGs in BLCA. First, we identified dysregulated protein-coding genes between Consensus cluster 1 and Consensus cluster 2 by differential expression analysis. Next, dysregulated genes between High and Low HP groups were identified. Then, we applied a Random Walk with Restart (RWR) based on a background protein–protein interaction network from a previous study [36]. Specifically, we project prognosis-related hypoxia genes to the PPI network as seed nodes for network propagation. Then, the RWR was used to explore the proximity between hypoxia genes and other genes. Genes with scores ranked in the top 10% after network propagation were considered as candidates (A similar ranking threshold has been applied in previous studies) [37–39]. We defined the intersection gene set of the above three sets as HRGs.

### Prediction of Potential Chemotherapy Drugs and Gene Targets for BLCA

We downloaded the gene expression profiles of BLCA tumor cell lines and the half maximal inhibitory concentration (IC50) values of chemotherapy drugs from Genomics of Drug Sensitivity in Cancer (GDSC) database (https://www.cancerrxgene.org/) [40]. We divided cell line samples into two groups based on the median value of each drug, considered as ‘sensitive’ and ‘resistant’ groups, respectively. Then, ‘limma’ v 3.54.2 was applied to identify dysregulated HRGs between sensitive and resistant groups (*P* < 0.05). Dysregulated HRGs were predicted to be the potential targets of certain drugs.

### Functional Enrichment analysis

Functional enrichment analysis was performed using the Metascape (https://metascape.org) platform [41]. GSEA enrichment was performed by R package ‘fgsea’ v 1.24.0.

### Construction of Risk Score Model by Drug–Target HRGs in BLCA

First, we performed univariate Cox regression analysis on the predicted targets of doxorubicin and identified OS-related HRGs in TCGA BLCA patients. Next, we constructed a nomogram to predict patients’ 1-, 3-, and 5-year OS probabilities based on OS-related HRGs. Calibration curves were used to evaluate the prediction power of the nomogram. Then, we constructed a Risk Score model based on the coefficients of multivariate Cox regression analysis and the expression levels of OS-related HRGs. The Risk Score calculation formula is as follows:

$$\begin{gathered} RiskScore = - \,0.0283 \times ACTG2 + 0.1704 \times MYC + 0.2600 \times PDGFRB \hfill \\ - 0.0444 \times DHRS2 - 0.1710 \times KLRD1 \hfill \\ \end{gathered}$$

The prediction power of the Risk Score model was tested in two independent cohorts GSE48075 and GSE31684 [42, 43] by comparing the survival differences between predicted high- and low-risk groups.

## Results

### Distinct Expression Patterns and Genetic Alterations of Hypoxia Genes in BLCA

**Fig. 1:**
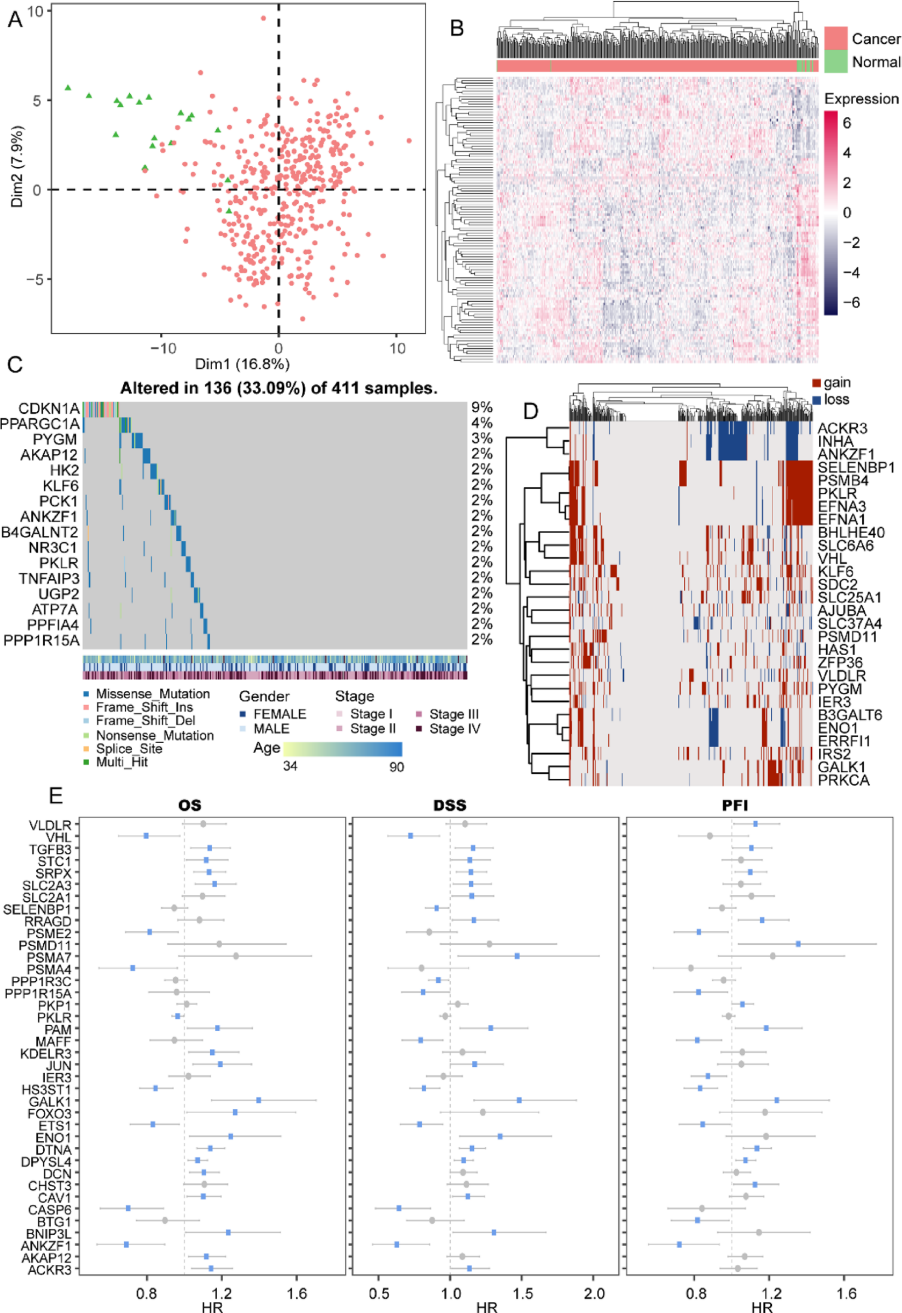
Expression and genomic variation of hypoxia genes in BLCA. **A** Principal component analysis between BLCA and normal samples based on the expression of dysregulated hypoxia genes. **B** Heatmap showing the expression of dysregulated hypoxia genes. **C** Somatic mutation map of hypoxia genes in BLCA samples. **D** Copy number variation of hypoxia genes in BLCA samples. **E** Three different survival analyses on hypoxia genes in BLCA samples (OS, DSS, PFI)

Deciphering the role of hypoxia genes in bladder cancer is essential to figuring out the key mechanisms underlying tumor initiation, progression, and metastasis. First, we performed differential expression analysis on 260 hypoxia genes based on the transcriptomic profiles of 411 bladder cancer (BLCA) samples and 19 corresponding normal controls from the TCGA database. As a result, 109 hypoxia genes showed dysregulated expression patterns in BLCA samples (limma fdr < 0.05). The results of principal component analysis (PCA) and hierarchical clustering analysis indicated a significant difference between BLCA, and normal samples based on the expression of dysregulated hypoxia genes, suggesting potential oncogenic roles of hypoxia genes (Fig. 1A, B). Next, we examined the genomic profiles of hypoxia genes in tumor samples. Several hypoxia genes with high-frequency somatic mutations were identified, including CDKN1A, PPARGC1A, and AKAP12 (Fig. 1C), which showed unequivocal associations with tumor initiation [44–46]. Copy number variation (CNV) analysis also revealed frequent amplifications and deletions of several important hypoxia genes in BLCA (Fig. 1D). Finally, we correlated hypoxia genes exhibiting differential expression between tumor and normal samples with patient survival data. Results suggested that most dysregulated genes displayed prognostic value in at least one type of patients’ survival (Fig. 1E). In summary, the integrated multi-omic data analysis suggested that hypoxia genes might play a pivotal role in the development and prognosis of bladder cancer, holding promise as potential markers to enhance our understanding of the disease.

### Two BLCA Clusters were Defined by Dysregulated Hypoxia Genes

**Fig. 2:**
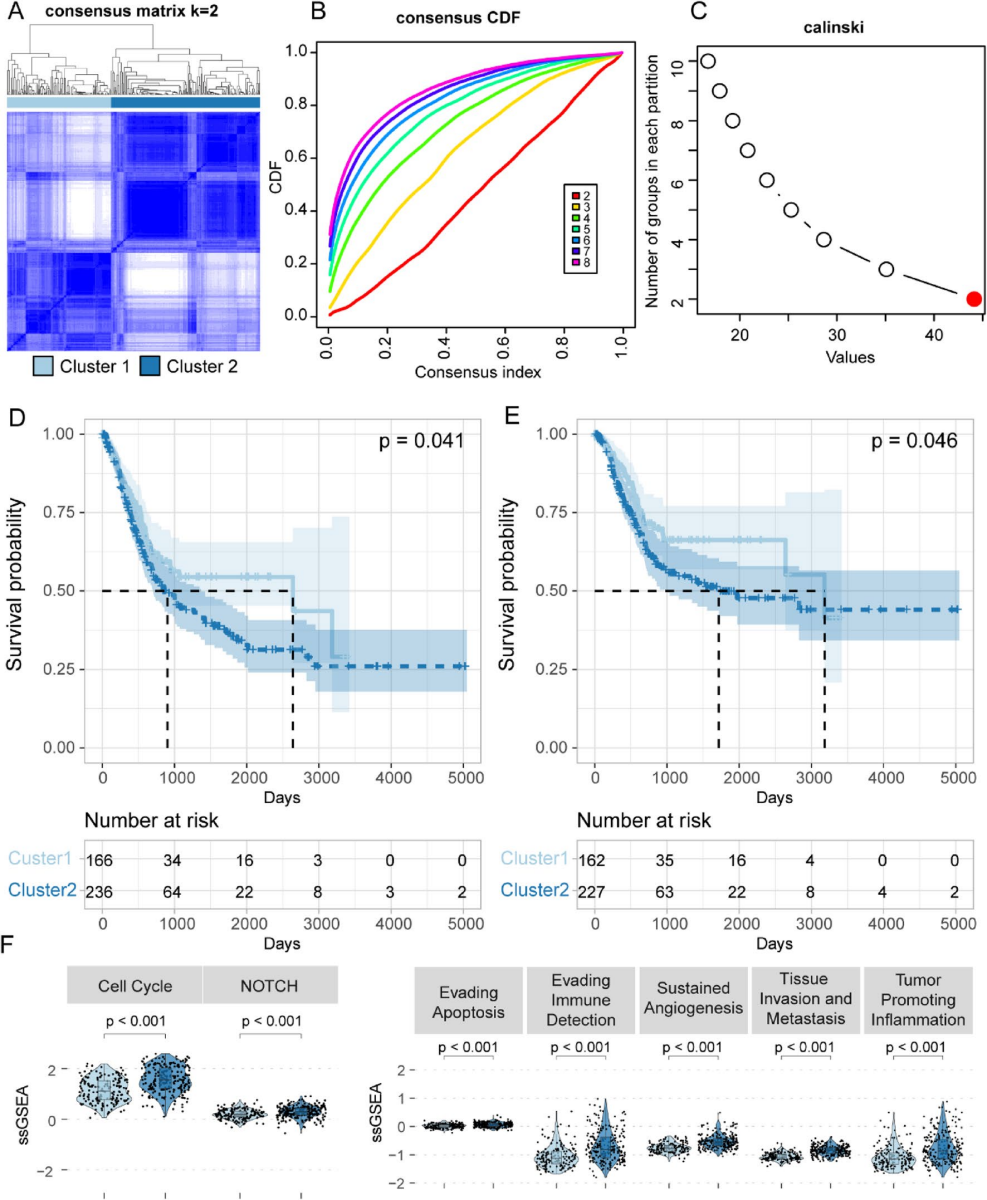
Identification of two BLCA clusters with distinct prognosis and oncogenic roles. **A** Two BLCA clusters identified by consensus clustering. **B** The relative change in the area under the CDF curve (from *k* = 2 to 8). **C** Optimal clustering k calculated by Calinski criterion. **D** Differences in OS between the two BLCA clusters. **E** Differences in DSS between the two BLCA clusters. **F** The violin plot illustrating the expression levels of oncogenic gene sets between BLCA and normal samples

Here, we conducted consensus clustering to cancer samples based on the 109 dysregulated hypoxia genes identified in Fig. 1B. The determination of the optimal number of clusters was executed through the Calinski criterion and the relative change in the area under the CDF curve (Fig. 2A–C). Consequently, we defined two distinct BLCA clusters: Cluster 1 encompassing 166 patients and Cluster 2 comprising 236 patients (Fig. 2A). Survival analysis suggested that patients from Cluster 1 had prolonged OS and DSS than patients from Cluster 2 (Fig. 2D, E). However, no significant PFI differences were found between the two clusters (Supplementary Fig. 1A). Given the potential role of hypoxia genes during BLCA development identified in Result 1, we sought to further investigate the oncogenic roles of hypoxia genes. Here, we quantified and compared the expression levels of 20 oncogenic gene sets between two clusters by ssGSEA method. Remarkably, Cell cycle, NOTCH signaling pathway, Evading Apoptosis, Sustained Angiogenesis, Tissue Invasion and Metastasis, and Tumor Promoting Inflammation gene sets were up-regulated in Cluster 2 (Fig. 2F). The elevation of these oncogenic pathways might contribute to the unfavorable survival outcomes observed in Cluster 2. In summary, our findings suggested a potential role of hypoxia genes during tumorigenesis.

### HPscore was Correlated with Clinical Features in BLCA

**Fig. 3:**
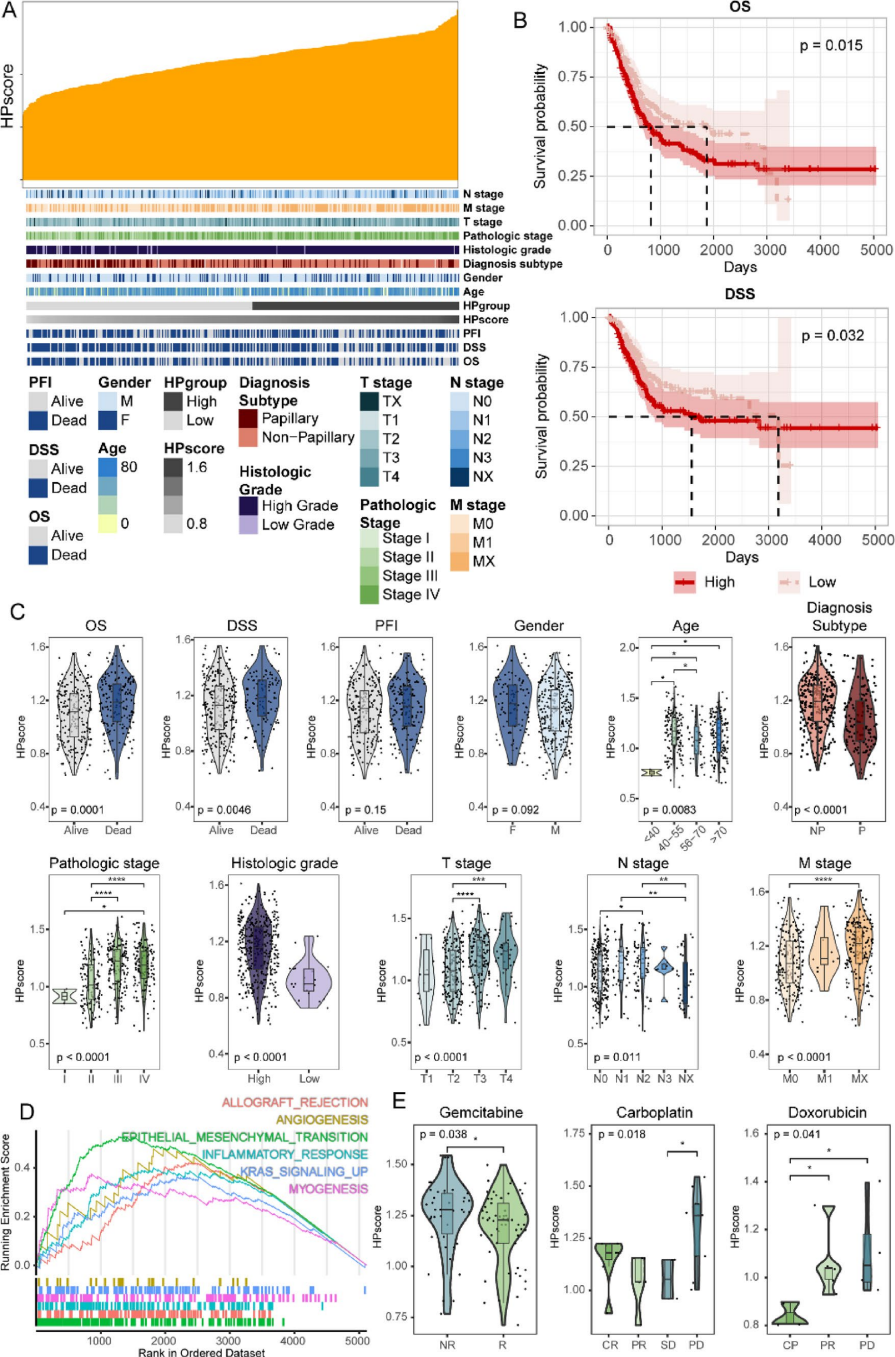
Correlations between HPscore and clinical characteristics in BLCA samples. **A** The correlations between HPscore and clinical characteristics, density curves show the HPscore for all cancer samples, as well as the corresponding clinical information (gender, age, etc.). **B** Survival differences between High and Low HP groups. **C** The violin plot illustrating the differences in the expression levels of HPscore among different groups of clinical characteristics. **D** GSEA analysis of dysregulated genes between High and Low HP groups. **E** Differences in HPscore among patients of distinct responses to three drug treatments

Here, we sought to interrogate the associations between clinical features and hypoxia genes (Fig. 3A). First, we defined a hypoxia score (HPscore) using the ssGSEA method based on the dysregulated hypoxia genes, which had an impact on the overall survival of BLCA patients. Next, we divided BLCA patients into two subgroups based on the median value of HPscore (High HP group, *n* = 192, Low HP group, *n* = 210). Survival analysis showed that patients with lower HPscore had better OS and DSS than those with higher HPscore, whereas PFI showed no significant differences between High and Low HP group (Fig. 3B, Supplementary Fig. 1B). Moreover, we explored the specific relationship between HPscore and other clinical features, including gender, age, diagnosis subtype, pathologic stage, histologic grade, and TNM stage. Notably, the results showed that higher HPscore were associated with higher TNM stage and pathologic grade. Meanwhile, the more malignant non-papillary subtype is also predominantly distributed in the High HP group (Fig. 3C). Regarding gender and age, the HPscore showed a complex changing pattern with age, and no significant differences were found between male and female patients. Additionally, we conducted differential expression analysis between samples from the High and Low HP groups, identifying 5120 dysregulated genes (Supplementary Fig. 1D). GSEA analysis was performed on these genes and results showed that angiogenesis, epithelial–mesenchymal transition (EMT), inflammatory response, and myogenesis were enriched in up-regulated genes in High HP group (Fig. 3D). These terms have been demonstrated to be associated with tumor progress [6, 47–49]. Finally, we investigated the potential relationship between HPscore and patients’ response to clinical treatments based on the chemotherapy response data from TCGA. As a result, elevated levels of HPscore were observed in non-responder than in responders after treatments of three drugs, including gemcitabine, carboplatin, and doxorubicin (Fig. 3E). Our results suggested a significant clinical relevance of HPscore, and a potential role of hypoxia genes in patients’ response to chemotherapy.

### Hypoxia’s Influence on Tumor Immune Landscape in BLCA

**Fig. 4:**
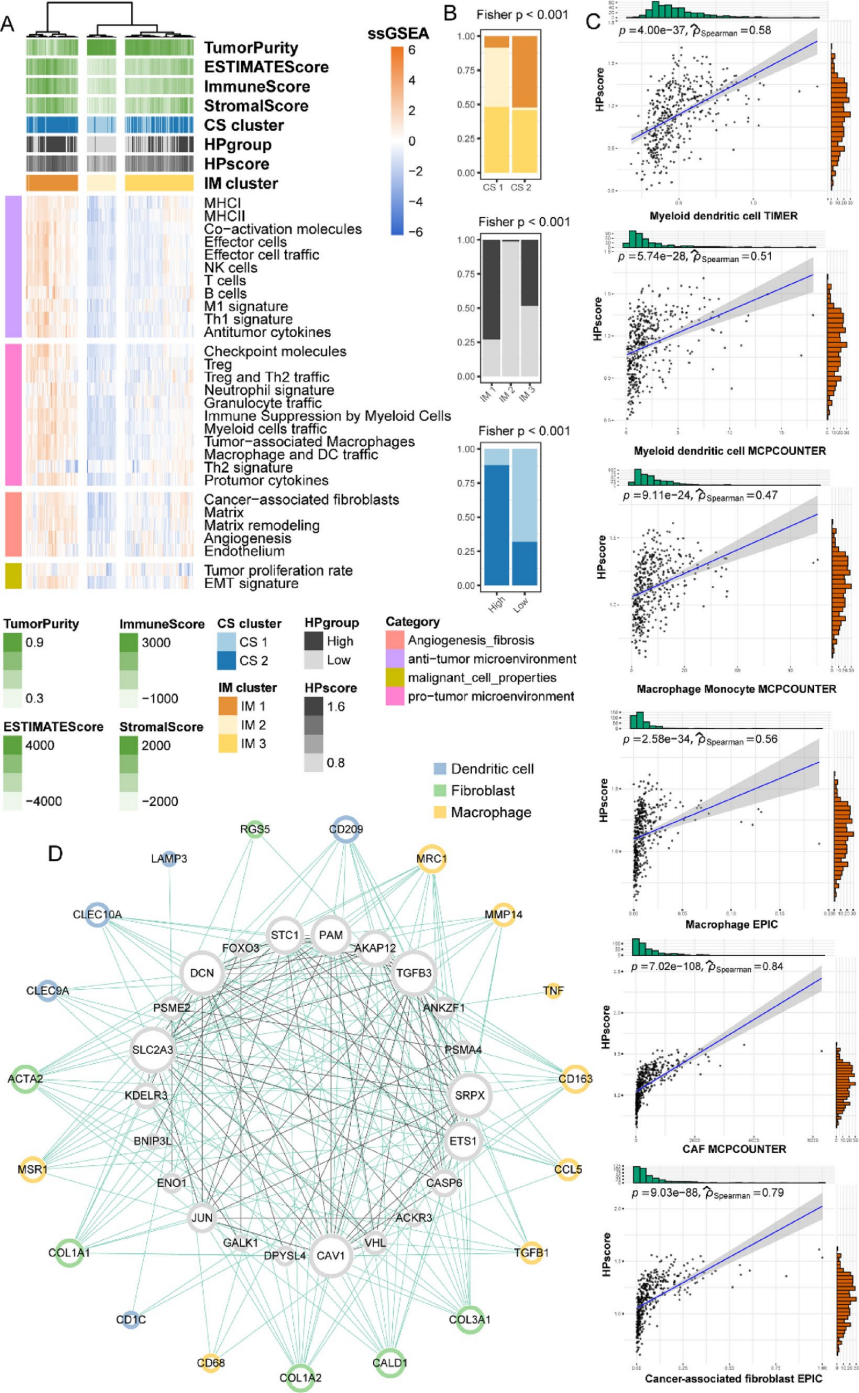
The role of hypoxia genes in BLCA TIME. **A** Heatmap showing three immune clusters of BLCA defined by 29 TIME features. **B** Stack bar plots showing the overlaps between consensus clusters, High/Low HP groups and IM clusters, respectively. **C** Correlation curves between HPscore and abundance of various immune cells. **D** Co-expression network of hypoxia genes and three immune-cell marker genes

Considering the results of the GSEA analysis, we aimed to explore the connection between the HPscore and the TIME. In a prior study, 29 knowledge-based gene expression features characterizing functional and cellular aspects of the TIME were defined. In this study, we assessed the enrichment levels of these 29 features in BLCA samples using ssGSEA. Subsequently, an unsupervised hierarchical clustering method was applied to the TIME features, identifying three distinct immune clusters (IM clusters) (Fig. 4A). Fisher’s test revealed significant overlap between IM clusters and the two consensus clusters (CS clusters) as well as the High/Low HP groups defined in Result 2 and Result 3 (Fig. 4B). IM1 showed a notably higher proportion of CS2 with a poor prognosis compared to CS1. IM1 exhibited the highest proportion of high HPscore among the three IM subgroups, while CS2 demonstrated the highest proportion of high HPscore subgroups, consistent with the earlier observation linking CS2 with poor prognosis and high HPscore. The above results elucidated that HPscore was recognized to impact the immune microenvironment of BLCA. Therefore, we computed the correlation between HPscore and the abundance of various immune cells, revealing a significant association with dendritic cells, macrophages, and fibroblasts (Fig. 4C). To further validate this result, we constructed a co-expression network between hypoxia genes and the expression of three immune cell subtypes surface marker genes and found close interactions between hypoxia genes and these three immune cell subtypes (Fig. 4D). In summary, the discernment of unique immune clusters and the correlation between immune cells and HPscore emphasized the significance of hypoxia genes in influencing the immune landscape in BLCA. These insights contributed valuable understanding to the intricate interactions within the tumor microenvironment.

### Unveiling Key HRGs Serving as Drug Targets in BLCA

**Fig. 5:**
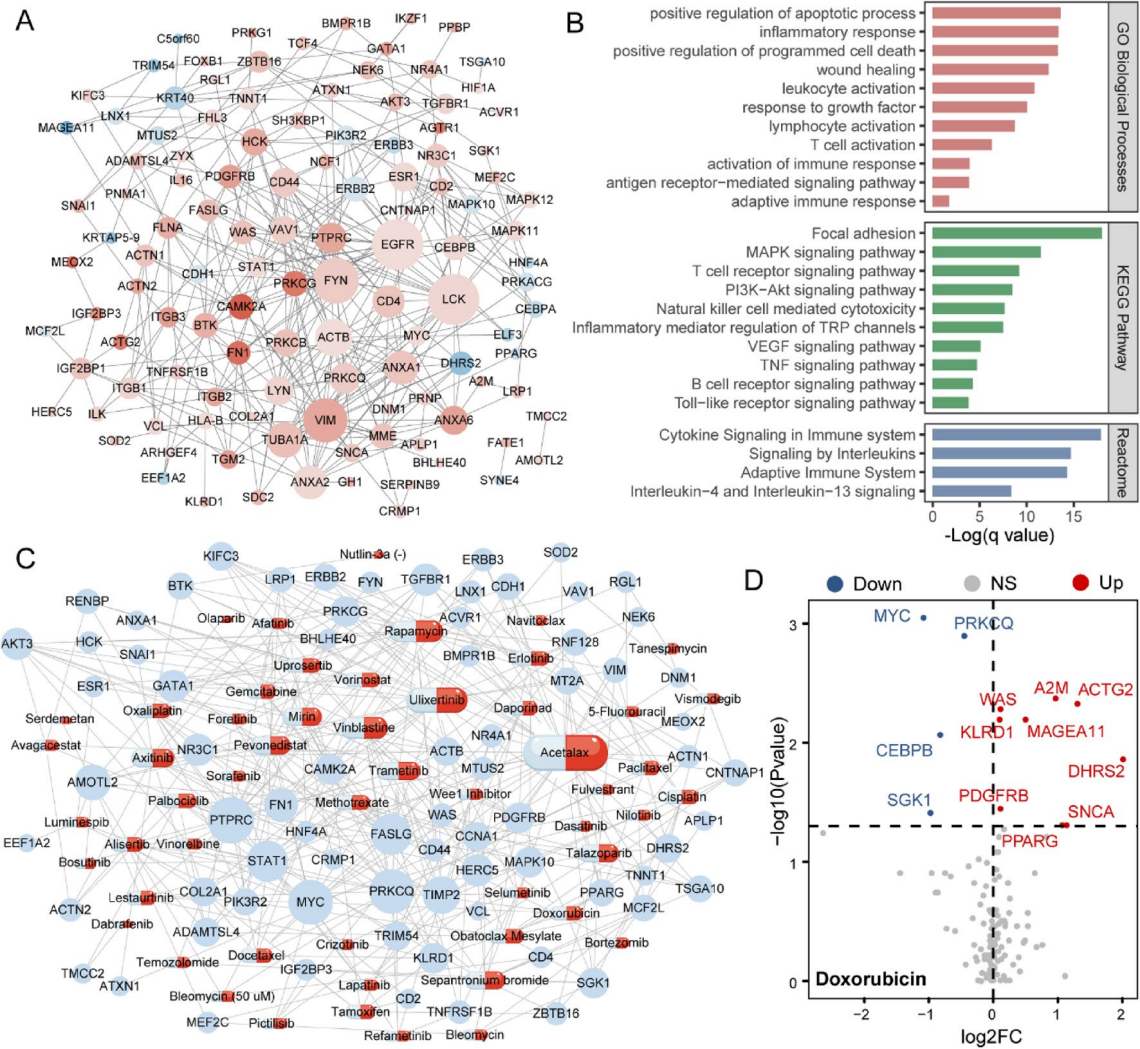
Unveiling key HRGs serving as drug targets in BLCA. **A** Network showing the HRGs identified, colors represent expression changing patterns in BLCA samples. **B** Functional enrichment analysis of HRGs (GO, KEGG, and Reactome). **C** Network showing predicted drug–target interactions. **D** Dysregulated HRGs between doxorubicin-sensitive and resistant- groups

To unveil the pivotal genes influencing the hypoxia-related process in BLCA, we employed the Random Walk with Restart (RWR) method, delving into a thorough exploration of essential HRGs. We identified the top 10% ranked genes of RWR as candidate genes and intersected them with DEGs identified in Result 2 and Result 3 (see Methods, Supplementary Fig. 1C, D). As a result, 129 HRGs were identified in BLCA (Fig. 5A). In continuation, a functional enrichment analysis showed that HRGs were enriched in GO biological processes, including positive regulation of the apoptotic process, positive regulation of programmed cell death and response to growth factor, indicating that HRGs could influence normal apoptosis while strongly promoting tumor development. HRGs also enriched in KEGG pathways including Focal adhesion, the MAPK signaling pathway, and the PI3K-Akt signaling pathways, which have long been demonstrated to have a strong correlation with the advancement of tumor (Fig. 5B) [50–52]. Given the correlations between HPscore and patients’ response to chemotherapy we identified in Result 3, we sought to further investigate whether and how HRGs could contribute to the drug resistance of BLCA. We categorized the tumor cell lines into sensitive and resistant groups based on the IC50 values of each drug. Following this categorization, we identified HRGs exhibiting distinct expression patterns between the two groups, leading to the construction of a comprehensive drug–target network (Fig. 5C). Within this network, we identified doxorubicin, an anthracycline-based chemotherapeutic agent demonstrating significant relevance in the network of resistance-associated genes. Our subsequent focus then centered on its 13 potential target HRGs, which exhibited perturbed expression patterns in the resistant group (Fig. 5D). Summing up, our results provided insights into potential candidate genes influencing hypoxia state in BLCA, offering potential implications for targeted therapies.

### Prognostic Significance of Doxorubicin-Target HRGs in BLCA

**Fig. 6:**
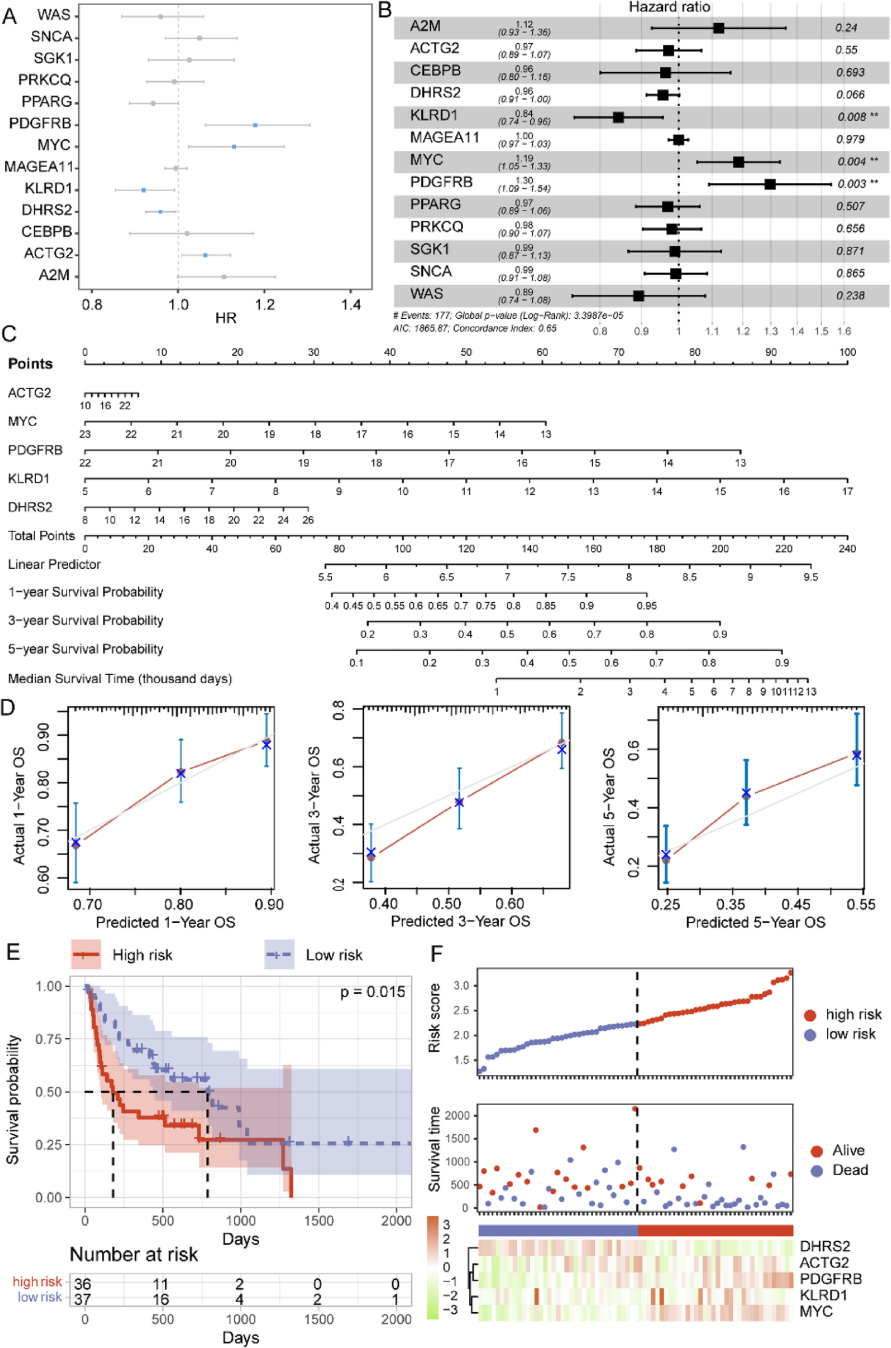
Prognostic value of doxorubicin-target HRGs in BLCA. **A** Forest plot showing the results of univariate Cox regression model of 13 doxorubicin-target HRGs. **B** Forest plot illustrating independent prognostic factors identified by the multivariate Cox regression model. **C** The nomogram showing the prediction model constructed based on five doxorubicin-target HRGs. **D** The calibration curves evaluating the predictive performance of the nomogram. **E** K–M curves showing the survival differences between predicted high- and low-risk groups in GSE48075 cohort. F. Correlation between survival time and expression levels of five HRGs

Using the 13 predicted target genes of doxorubicin, we performed a univariate Cox analysis based on TCGA BLCA samples (Fig. 6A). Elevated expressions of PDGFRB, MYC, and ACTG2 were linked to poorer OS in BLCA patients. KLRD1 and DHRS2 were identified as protective factors for BLCA prognosis. Subsequently, a multivariate Cox regression model was applied to identify independent prognostic factors. Among the target genes, only MYC, KLRD, and PDGFRB retained significant associations with patients’ OS (Fig. 6B). Consequently, we developed a nomogram based on the five OS-related genes of univariate Cox regression. The nomogram aimed to predict 1-, 3-, and 5-year survival probabilities, as well as the median survival time for BLCA patients (Fig. 6C). Calibration plots were then utilized to assess the predictive accuracy of the nomogram. The results revealed minimal disparities between predicted and observed overall survival, indicating the good predictive accuracy of the nomogram (Fig. 6D). Then, we constructed a Risk Score model based on the expression of the five doxorubicin-target genes using the TCGA BLCA dataset. To evaluate the robustness and generalizability of the Risk Score model, we collected two independent BLCA cohorts from the GEO database to validate the predictive power of the model. The high-risk group exhibited a significantly poorer prognosis compared to the low-risk group (Fig. 6E, Supplementary Fig. 2A). Finally, we ranked GEO patients based on their Risk Score, simultaneously illustrating the correlation between their survival time and the expression levels of the five candidate genes (Fig. 6F, Supplementary Fig. 2B). Our results identified potential drug resistance mechanism of BLCA by HRGs and suggested a prognosis value of doxorubicin-target genes.

## Discussion

Hypoxia, prevalent in the majority of solid tumor [53], arises from an imbalance between heightened oxygen demand and inadequate supply, attributed to the rapid proliferation of tumor cells [54, 55]. This hypoxic milieu exerts a profound influence on diverse biological processes, including metabolism, angiogenesis, and metastasis [56–58]. A previous study has established a correlation between the hypoxia-related HIF-1α signaling pathway and the development of bladder cancer [59]. However, a comprehensive investigation of hypoxia genes in bladder cancer, particularly through large-scale multi-omic data analysis, remains largely unexplored. In this study, we extensively investigated hypoxia genes in bladder cancer, exploring their transcriptomic and genomic alterations, clinical implications, and correlation with the TIME. Intriguing outcomes were found during the analysis of genomic data. Previous studies have highlighted the involvement of PYGM, a glycogen phosphorylase isoform, in bladder cancer through its role in glycogen breakdown, essential for cancer cell survival and adaptation to low oxygen conditions [60, 61]. However, we observed a high frequency of somatic mutations and copy number amplifications in the PYGM gene in bladder cancer patients. These findings provided evidence for the potential contribution of PYGM alterations to bladder cancer pathogenesis.

Previous studies have attempted to define bladder cancer subtypes using various molecular subtyping systems, but their clinical significance remains uncertain [62]. Our study employed dysregulated hypoxia genes with prognostic values to define bladder cancer subtypes, offering valuable insights into bladder cancer heterogeneity and potential implications for personalized treatment strategies. The introduction of the HPscore serves as a powerful tool, linking differential gene expression to clinical features. Similar methods have been used to characterize hypoxia status in immunosuppression of thyroid cancer [63]. This scoring system not only aids in prognostic assessments but also opens avenues for personalized treatment approaches. Our HPscore not only holds prognostic significance but also offers potential indications for personalized treatment strategies, particularly in relation to chemotherapy response and the modulation of the TIME. Previous studies have extensively investigated the impact of hypoxia on chemotherapy resistance in bladder cancer, particularly with cisplatin and gemcitabine [64–66]. These studies have highlighted the role of hypoxia-induced pathways in promoting resistance to these chemotherapeutic agents. Our study employed a network-based approach to identify HRGs with prognostic implications that could serve as potential therapeutic targets of doxorubicin treatment in bladder cancer. These results uncovered the molecular mechanisms that contribute to chemotherapy response in bladder cancer, presenting potential avenues for personalized treatment strategies.

Although our research identified the potential of HRGs as treatment targets for doxorubicin in bladder cancer, it is important to note that further experiments and validations are required to enhance the robustness of the results. The findings of our work provide preliminary candidates for future studies, serving as a foundation for exploring the therapeutic potential of doxorubicin in bladder cancer. Despite these limitations, this study represents a significant stride in unraveling the complexities of bladder cancer. These findings established a groundwork for further exploration and development of personalized therapeutic strategies for patients with bladder cancer.

## Conclusion

This study investigated the role of hypoxia genes in bladder cancer and their impact on chemotherapy response. By analyzing transcriptomic and genomic data from 411 bladder cancer samples, we identified dysregulated hypoxia genes and delineated two distinct bladder cancer clusters with different survival outcomes. This study also revealed unique associations between hypoxia genes and TIME, highlighting the influence of hypoxia on the immune landscape in bladder cancer. Additionally, using a network-based approach, this study identified HRGs that could serve as potential targets for doxorubicin treatment in bladder cancer, offering insights into potential avenues for targeted therapies. Overall, this study enhanced our understanding of bladder cancer at the molecular level and provided potential opportunities for personalized treatment strategies. The identification of HRGs as prognostic indicators and potential therapeutic targets represents a significant advancement in bladder cancer research.

## Supplementary Information

Below is the link to the electronic supplementary material.

Supplementary file1 (PDF 761 KB)

Supplementary file2 (DOCX 24 KB)

## Data Availability

The datasets generated and/or analyzed during the current study are available in the Xena Browser repository at https://xenabrowser.net/.
