# Supplementary figures and images for: Predicting the Prognosis of Bladder Cancer Patients Through Integrated Multi-omics Exploration of Chemotherapy-Related Hypoxia Genes
Source: Mol Biotechnol. 2024 May 28;67(6):2367–81. doi: 10.1007/s12033-024-01203-9 (PMC12055635; doi:10.1007/s12033-024-01203-9)

**A**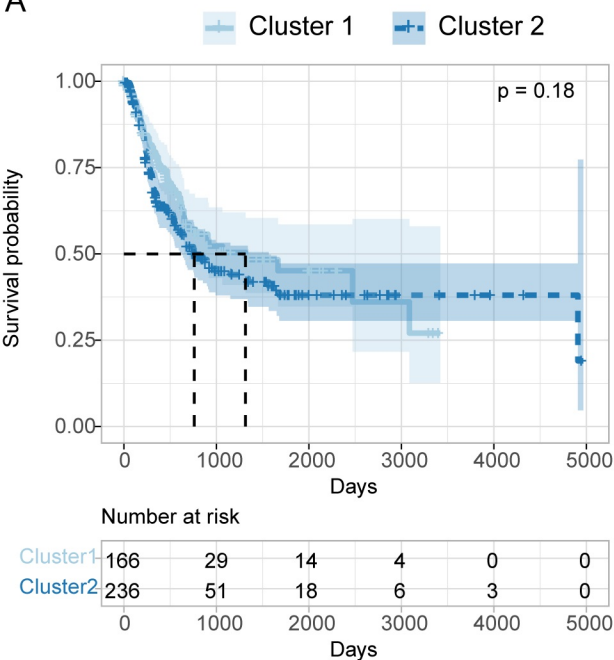**B**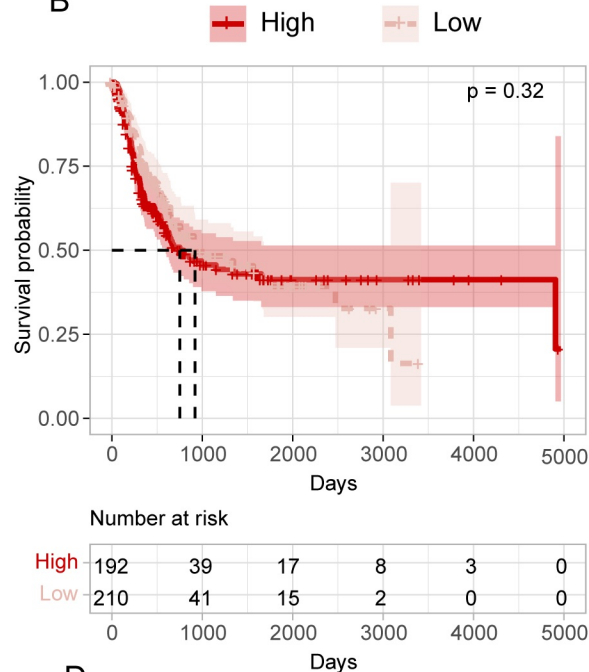**C**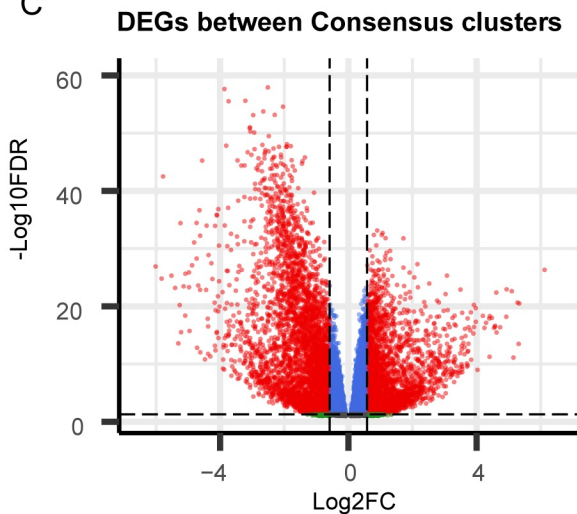**D**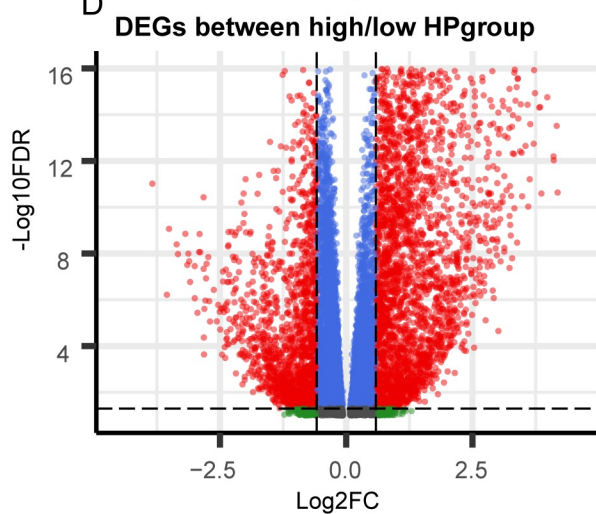

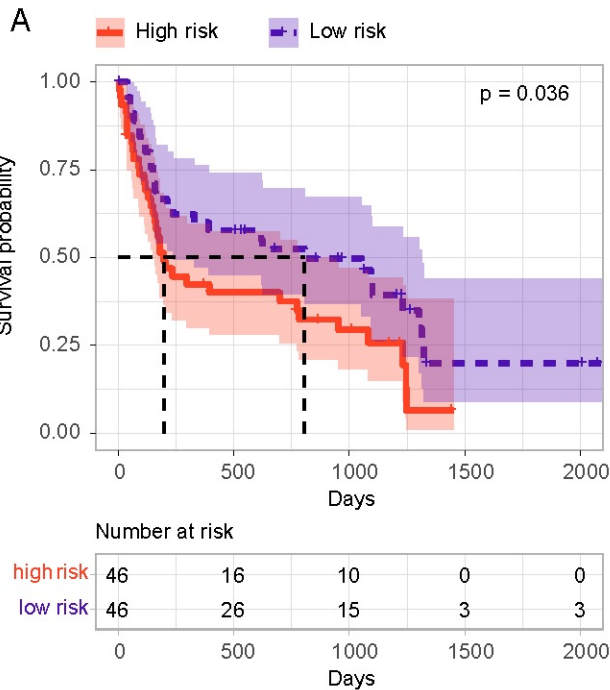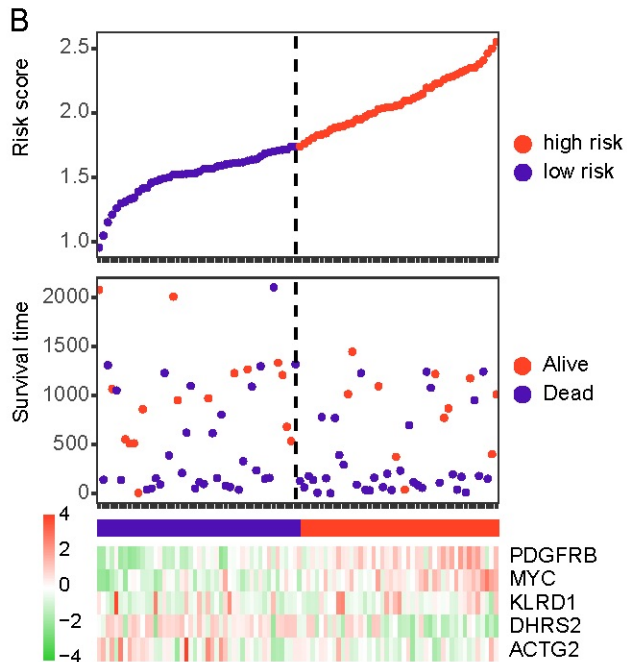

Supplement: Supplementary file 1 — Supplementary file1 (PDF 761 KB) [file 12033_2024_1203_MOESM1_ESM.pdf]
