# Supplementary material for: Predicting the Prognosis of Bladder Cancer Patients Through Integrated Multi-omics Exploration of Chemotherapy-Related Hypoxia Genes
Source: Mol Biotechnol. 2024 May 28;67(6):2367–81. doi: 10.1007/s12033-024-01203-9 (PMC12055635; doi:10.1007/s12033-024-01203-9)
Supplement: Supplementary file 2 — Supplementary file2 (DOCX 24 KB) [file 12033_2024_1203_MOESM2_ESM.docx]

**Supplementary Fig. 1**

A. Survival analysis of PFI between two clusters. B. Survival analysis of PFI between High and Low HPgroups. C. Differential expression genes between consensus cluster 1 and cluster 2. D. Differential expression genes between from High and Low HPgroups.

**Supplementary Fig. 2 Risk score model validation in GSE31684**

A. KM curves showing the survival differences between predicted high- and low- risk groups in GSE31684 cohort. B. Correlation between survival time and expression levels of five HRGs.
